# Annotations

**Published:** 1905-04-22

**Authors:** 


### ANNOTATIONS.

### The Typhoid Epidemic at Lincoln.

We are glad to learn that the epidemic of typhoid fever at Lincoln has taken a turn for the better, and that there is every reason to hope that the figures at the end of April will show a marked improvement on those of February and March. This is all the more satisfactory because alarmist statements have been current, owing perhaps to the notification of 24 new cases in four days during the present month. But on other days, previously and since, the number has been considerably smaller, and on the 13th there was not one. The seven hospitals in the city are still open, and are all practically filled, but in consequence of the decrease of cases it is now much easier to meet the demand for beds than it was a few weeks ago. Convalescent patients are daily being drafted to Convalescent Homes, both from the hospitals and from the houses of the poor. Some of these are sent to homes provided by the Mayor's Relief Fund, and others to homes provided by Miss Bromhead's Patients' Relief Fund. It is, however, necessary to add that the epidemic is by no means at an end, and that it is absolutely essential to observe the most stringent precautions. The cost of the outbreak is likely to be very serious. Already the City Council have expended £4,000, and it is expected that about £5,000 more will be needed, supposing that there is no recrudescence of the epidemic. In addition to this, the trade of Lincoln has been gravely affected. The lesson will not, however, have been experienced in vain if the authorities do their duty in respect to the permanent water supply, and the citizens are able to feel that henceforth they can venture to drink it without the fear of being prostrated by a disease which is so clearly preventable.

### Lunacy Acts Amendment (London) Bill.

Under this title there has been presented to Parliament a Bill which may be said to recognise the fact that by the proper treatment of mental disease in its earlier stage certain persons who, under existing arrangements, are usually sent to a county lunatic asylum after examination in a workhouse may be saved from the stigma of lunacy. Hence it is proposed to enable the London County Council to establish houses at which persons alleged to be lunatics may be received for examination and treatment, each of such houses to be in charge of a medical officer as superintendent. Detention in the receiving house will require the authority of a justice of the peace, who must, before giving his sanction, take the opinion of a medical practitioner and make such other inquiries as he may think advisable. Provision may also be made for the treatment as out-patients of persons mentally affected, and neither these, nor those detained under a detention order, shall be deemed to be lunatics. Persons who, under the Lunacy Acts, 1890 and 1891, would be removed to a workhouse, or an inmate of a workhouse alleged to be of unsound mind, will under the proposed Bill be conveyed to a receiving house, and may, after examination by the superintendent, be detained there for a period not exceeding three days. But before the expiration of that period such persons shall either be discharged or brought before a justice of the peace, who, after hearing the report of the superintendent, may issue a detention order or, alternatively, may proceed as under the present regulations. The provisions which apply to the supervision and visitation of lunatic asylums are to apply generally to the proposed reception houses, each of which shall be visited at least once a year by one or more of the Commissioners in Lunacy. Presuming that the scheme here outlined comes into operation an interesting experiment will be inaugurated. That this will be attended by success can hardly be doubted, seeing that of 261 pauper lunatics admitted to an "observation ward" in Glasgow in 1903, 219 were, after a period of detention, restored to the privilege of civil freedom. Most of these, in other circumstances, would have at once been sent to a lunatic asylum.

### The Pharmaceutical Society and the Poisons Schedule.

Amongst other statutory duties imposed on the Council of the Pharmaceutical Society is the responsibility of determining, subject to the sanction of the Privy Council, the substances which shall come under the legal restrictions which govern the sale of poisons. According to those restrictions, "poisons" can only be sold by a person registered under the Pharmacy Act (though this clause does not apply to medical practitioners), and they must be properly labelled and bear the name and address of the seller. In addition, the sale of certain of them having specially virulent properties must be registered in a book kept for the purpose. The Council has now recommended that this latter group shall be extended by the addition of cocaine and its salts and of picrotoxin; also that a number of other substances, including acetanilide, sulphonal, preparations of digitalis, of strophanthus and of cocaine, and certain salts of mercury and oxalic acid shall come under the more general regulations. Assuming that the Privy Council endorses the Council's recommendation this will at once have legal force, and we believe to the advantage of the public. The only objection is likely to come from traders who are non-pharmacists and from manufacturers of proprietary medicines who use any of the above in their preparations. The latter will now be compelled to label each package with the name of the "poison" it contains, and hence the public will no longer purchase and take, without warning, a number of powerful and even dangerous drugs under alluring titles evoked by the stimulus of commercial ambition. The restriction of the sale of active medicinal agents to pharmacists, though it may savour of monopoly, is equally, we think, in the public interest. It at least means that these agents are distributed to the public through the hands of those who are able to appreciate the responsibility involved in such a proceeding, and it helps to place such substances in their true character before those who, wisely or unwisely, consider themselves able to prescribe for their own ailments.

